# Staphylococcal Protein A Induces Leukocyte Necrosis by Complexing with Human Immunoglobulins

**DOI:** 10.1128/mBio.00899-21

**Published:** 2021-06-01

**Authors:** Proinnsias G. Fox, Francesca Schiavetti, Rino Rappuoli, Rachel M. McLoughlin, Fabio Bagnoli

**Affiliations:** a GSK, Siena, Italy; b Host-Pathogen Interactions Group, School of Biochemistry and Immunology, Trinity Biomedical Sciences Institute, Trinity College Dublin, Dublin, Ireland; Pasteur Institute

**Keywords:** B cells, staphylococcal protein A, *Staphylococcus aureus*, vaccine

## Abstract

One of the defining features of Staphylococcus aureus is its ability to evade and impair the human immune response through expression of staphylococcal protein A (SpA). Herein, we describe a previously unknown mechanism by which SpA can form toxic immune complexes when in the presence of human serum, which leads to the loss of human leukocytes. Further, we demonstrate that these toxic complexes are formed specifically through SpA’s interaction with intact human IgG and that, in the presence of purified IgG Fab and Fc fragments, SpA shows no such toxicity. The mechanism of action of this toxicity appears to be one mediated by necrosis and not by apoptosis, as previously hypothesized, with up to 90% of human B cells rapidly becoming necrotic following stimulation with SpA-IgG complexes. This phenomenon depends on the immunoglobulin binding capacity of SpA, as a nonbinding mutant of SpA did not induce necrosis. Importantly, immune sera raised against SpA had the capacity to significantly reduce the observed toxicity. An unprecedented toxic effect of SpA-IgG complexes on monocytes was also observed, suggesting the existence of a novel mechanism independent from the interaction of SpA with the B cell receptor. Together, these data implicate SpA in inducing indiscriminate leukocyte toxicity upon formation of complexes with IgG and highlight the requirement for vaccination strategies to inhibit this mechanism.

## INTRODUCTION

Staphylococcus aureus infection represents one of the largest health care threats faced by humankind, with a reported mortality rate within the United States greater than that of HIV/AIDS, tuberculosis, and viral hepatitis combined (1). The burden of S. aureus on national health systems is expected to rise over the coming years as antimicrobial resistance strains continue to spread, with few treatment modalities being left open to clinicians (2–4). Despite several high-profile attempts and the obvious overwhelming health care need, there are currently no approved vaccine therapeutics for S. aureus (5, 6). One of the greatest impediments to the generation of an efficacious S. aureus vaccine has been the plethora of immune evasion factors and strategies employed by the pathogen, which have been shown to disrupt most, if not all, normal immunological responses. Of note is the family of toxins referred to as the superantigens (7, 8), which act to disrupt host adaptive immune responses via their noncanonical interactions with the primary activating complexes on either B or T cells. Staphylococcal protein A (SpA) is often presented as a model B cell superantigen, and it is thought to be one of the primary reasons why repeated exposure to S. aureus fails to generate protective humoral immunity (9).

SpA is an ∼45-kDa secreted and cell wall-associated virulence factor expressed in 93% of clinical S. aureus isolates that disrupts the humoral immune response (10–13). It consists of three regions: the immunoglobulin binding region, the X region, and a peptidoglycan tail (14, 15). The immunoglobulin binding region is responsible for the bulk of SpA’s biological effects, such as its binding (i) to the Fc portion of human IgM, IgD, and class I, II, and IV IgGs (16, 17), (ii) to the fragment antigen binding (Fab) region of V_H_3^+^ antibodies and B cell receptors (BCRs) (18, 19), (iii) to tumor necrosis factor receptor 1 (TNFR1) (20), (iv) to epithelial growth factor receptor (EGFR) (21), and (v) to von Willebrand factor (vWF) (8, 22). SpA has numerous interactions with and effects on the immune system; the best known and most salient of these effects are on the humoral immune response. SpA has been shown to sequester antibodies via their Fc domain, which reportedly orients the antibodies at an incorrect angle, thus strongly inhibiting opsonophagocytosis (12, 23). This inhibition of opsonophagocytosis is believed to be one of the primary contributing factors behind the poor efficacy of the antibody-mediated immune response to S. aureus (9). Indeed, most individuals are positive for staphylococcal antibodies, but these antibodies provide little protection against recurrent infection (24).

SpA’s B cell superantigen activity is predicated upon its ability to bind to and activate V_H_3^+^ BCRs via its interactions with the highly conserved CDR2 region (8, 25). These noncanonical Fab interactions allow SpA to interact with ∼30% of human peripheral B cells, compared to the expected 0.001 to 0.1% found with general antigens. On a cellular level, the biological consequence of this superantigen activation is not well understood. Initial experiments with mice using purified recombinant SpA demonstrated a marked loss of B cells and the formation of B220^+^ apoptotic bodies, indicating that SpA treatment caused the clonal collapse of activated B cell populations (26). It was later found that SpA produced recombinantly in Escherichia coli was lacking in the normally present peptidoglycan tail (27). Mice treated with full-length SpA produced natively in S. aureus demonstrated enhanced B cell activation, expansion, and antibody expression in a T cell-dependent manner (15). Mice, however, have much lower sensitivity to SpA, as they do not express the V_H_3 domain; instead, they express the homologous S107, J606, 7183, and DNA4 genes, and only ∼3 to 5% of mature mouse B cells display this nonimmune Fab-mediated SpA binding (26). In partial agreement with these findings, an *in vitro* study on human peripheral blood mononuclear cells (PBMCs) using recombinant SpA made in E. coli but used in concert with a Toll-like receptor 2 (TLR2) agonist also led to B cell expansion but failed to induce antibody expression; this TLR-dependent effect, however, was found to be T cell independent (28).

A recent study looking at the effects of SpA on murine B cell maturation and development using an S. aureus *spa* knockout strain (RN4220) found that SpA expression disrupted the generation of S. aureus-specific long-lived plasma cells (LLPCs) (29), which reportedly generate the bulk of circulating antibodies during secondary infections (30). However, the identification of large numbers of circulating S. aureus-specific human memory B cells and circulating V_H_3^+^ plasma cells postinfection (11, 24) have further conflated the issue.

Additionally, the ability of SpA to bind antibodies by both their Fc and Fab domains has been implicated in the generation of large immune complexes, which have been shown to have strong *in vitro* proinflammatory effects, e.g., neutrophil recruitment, mast cell activation, and complement activation (18). When administered in the presence of human serum or human IgG, SpA has been shown to initiate immune complex-dependent Arthus reactions in rabbits and mice (18, 31). A recent study by Ulloa-Morales et al. (32) expanded upon these findings by demonstrating that in the presence of human IgG, SpA can deplete up to 70% of the peripheral B cells found in a V_H_3^+^ T15i transgenic knock-in mouse model. This transgenic model also showed that anaphylaxis could be induced in mice pretreated with human IgG through exposure to 1 mg of SpA. This anaphylaxis and or B cell depletion has never been directly observed in humans. A wild-type version of the protein (PRTX‐100) developed by Protalex has been tested in six clinical trials at high dosages (i.e., 12 μg/kg of body weight) for assessing its use as a therapy against autoimmunity (33–35, 36), and it was found to be generally safe and well tolerated.

SpA’s ability to manipulate the humoral memory immune response and thus, by extension, its ability to interfere with vaccination efficacy has led to the development of a stable SpA mutant (SpA_MUT_), which has been shown to generate protective immunity when used in several animal models (10, 14, 16, 37). This mutant was generated through the introduction of specific mutations within the immunoglobulin binding domains of SpA that have been shown to convey protective effects in both active and passive vaccination experiments using animal models (14). Indeed, vaccination strategies using this mutant have been found to confer protection to S. aureus challenge in murine and guinea pig models as well as to increase decolonization rates of persistently colonized mice (14, 16, 38). These studies, however, did not to identify whether these protective effects might be reflective of the human response.

Within the present study, we investigate SpA’s role as a human B cell toxin in an *in vitro* setting, demonstrating for the first time that SpA can induce necrosis in target human cells in the presence of intact human IgG. We also show that this toxigenic effect is not limited to human B cells and that other leukocytes are also affected. Further, we demonstrate that passive vaccination with sera generated against a nonbinding mutant of SpA can inhibit this toxigenic interaction, offering significant protection to vulnerable human B cells. These data support the use of SpA_MUT_ as a possible vaccination target.

## RESULTS

### SpA’s activation of a human B cell line is inhibited by FBS.

**FIG 1:**
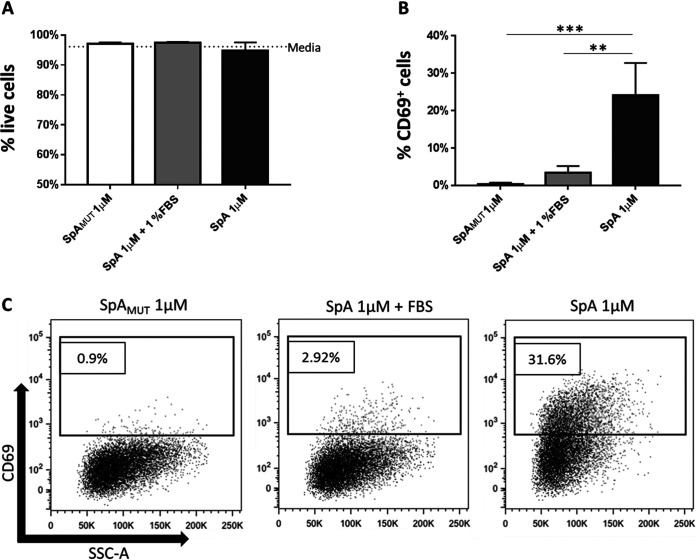
Activation of the human monoclonal B cell line MAVER-1 by SpA. MAVER-1 B cells (5 × 10^5^) were cultured for 2 h in media with and without supplementation with 1% FBS and either SpA or SpA_MUT_ (1 μM). (A) Live cells were identified by flow cytometry as morphologically distinct single cells that were negative for fixable near-infrared (near-IR) dead-cell stain. (B) Cells were then gated on CD69^+^ events and normalized by subtracting the medium background. (C) Representative dot plots showing cellular CD69 expression. *n* = 5. SSC-A, side scatter A. Data were analyzed by one-way ANOVA using Tukey’s multiple-comparison correction. ***, *P* < 0.0005; **, *P* < 0.005.

There have been several conflicting reports regarding the proposed effects of SpA on B cell responses, ranging from the expansion of human V_H_3^+^ B cell populations *in vitro* to the clonal collapse of specific mouse B cell populations when SpA is used *in vivo* (15, 26). The goal of this study was to identify whether stimulation with SpA leads to activation-induced apoptosis or expansion of human B cells. To this end, we established an *in vitro* assay to measure both cellular viability and expression of the activation marker CD69 by a human mantle V_H_3^+^ B cell lymphoma cell line (MAVER-1) (39). To control for non-BCR-mediated activation, we utilized a mutated variant of SpA (SpA_MUT_) (14). Even when used at a relatively high dose, 1 μM (42 μg/ml), stimulation with nonmutated SpA or SpA_MUT_ demonstrated no significant effect on cellular viability (Fig. 1A). Expression of the early activation marker CD69 by the MAVER-1 cells was, however, significantly increased following stimulation with 1 μM SpA for 2 h, in contrast to what occurred after stimulation with SpA_MUT_, which did not significantly increase CD69 expression over that of control cells in medium (Fig. 1B and C). Interestingly, it appeared that the presence of fetal bovine serum (FBS) within the stimulating medium inhibited cellular activation, with a >80% reduction in CD69 expression observed between the cells that were exposed to SpA in the presence of 1% FBS and the cells that were exposed to SpA in medium alone (Fig. 1B and C). The presence of FBS had no effect on cellular viability (Fig. 1A).

### SpA is toxic in the presence of human serum.

**FIG 2:**
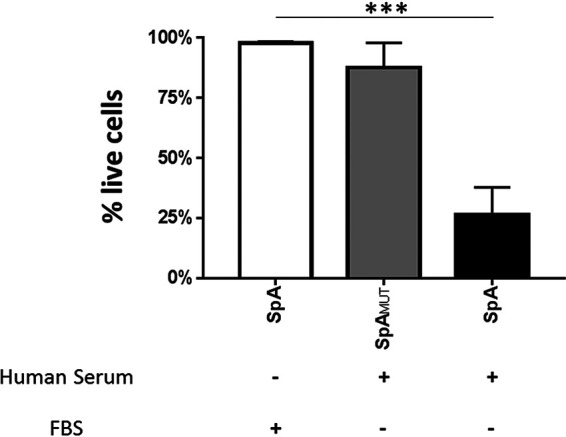
SpA is toxic to MAVER-1 cells in the presence of human serum. MAVER-1 cells (5 × 10^5^) were cultured for 2 h in media containing 1 μM SpA or SpA_MUT_ and supplemented with either 10% FBS or 10% human serum from a single donor. Live cells were identified by flow cytometry as morphologically distinct single cells that were negative for fixable near-IR dead-cell stain. *n* = 3. Results were analyzed by one-way ANOVA using Dunnett’s multiple-comparison test. ***, *P* < 0.0005.

Having demonstrated an inhibitory effect of FBS on SpA-mediated activation of the MAVER-1 B cell line, the impact of human serum on SpA-mediated activation of B cells was established. MAVER-1 cells were stimulated with either SpA or SpA_MUT_ in medium supplemented with 10% (vol/vol) human serum derived from a single healthy donor. When cultured in the presence of human serum, SpA induced significant cellular toxicity. This toxicity resulted in an average loss of ∼75% of the MAVER-1 B cells within 2 h (Fig. 2). No toxicity was observed in response to stimulation with SpA_MUT_, indicating that the observed toxicity is reliant upon the immunoglobulin binding domains present on SpA. This toxicity was also found to be reliant on the V_H_3 interaction, as V_H_3^–^ RAJI cells (40) were unaffected by SpA-mediated toxicity (Fig. S1). SpA was found to be equally toxic when used in conjunction with either 10% or 100% human serum (data not shown); thus, a concentration of 10% human serum was used, as it was the minimum concentration required to see a robust and reproducible effect.

10.1128/mBio.00899-21.1 FIG S1 The V_H_3^–^ B cell line RAJI is unaffected by SpA-mediated toxicity. RAJI and MAVER-1 cells (5 × 10^5^) were cultured for 2 h in medium containing 1 μM SpA and supplemented with 10% human serum from a single donor. Live cells were identified by flow cytometry as morphologically distinct single cells that are negative for fixable near-infrared (IR) dead-cell stain. Images are corrected for medium controls. Results from two independent experiments (mean values with SD) are shown. Download FIG S1, TIF file, 0.7 MB. Copyright © 2021 Fox et al. 2021 Fox et al. https://creativecommons.org/licenses/by/4.0/ This content is distributed under the terms of the Creative Commons Attribution 4.0 International license.

**FIG 3:**
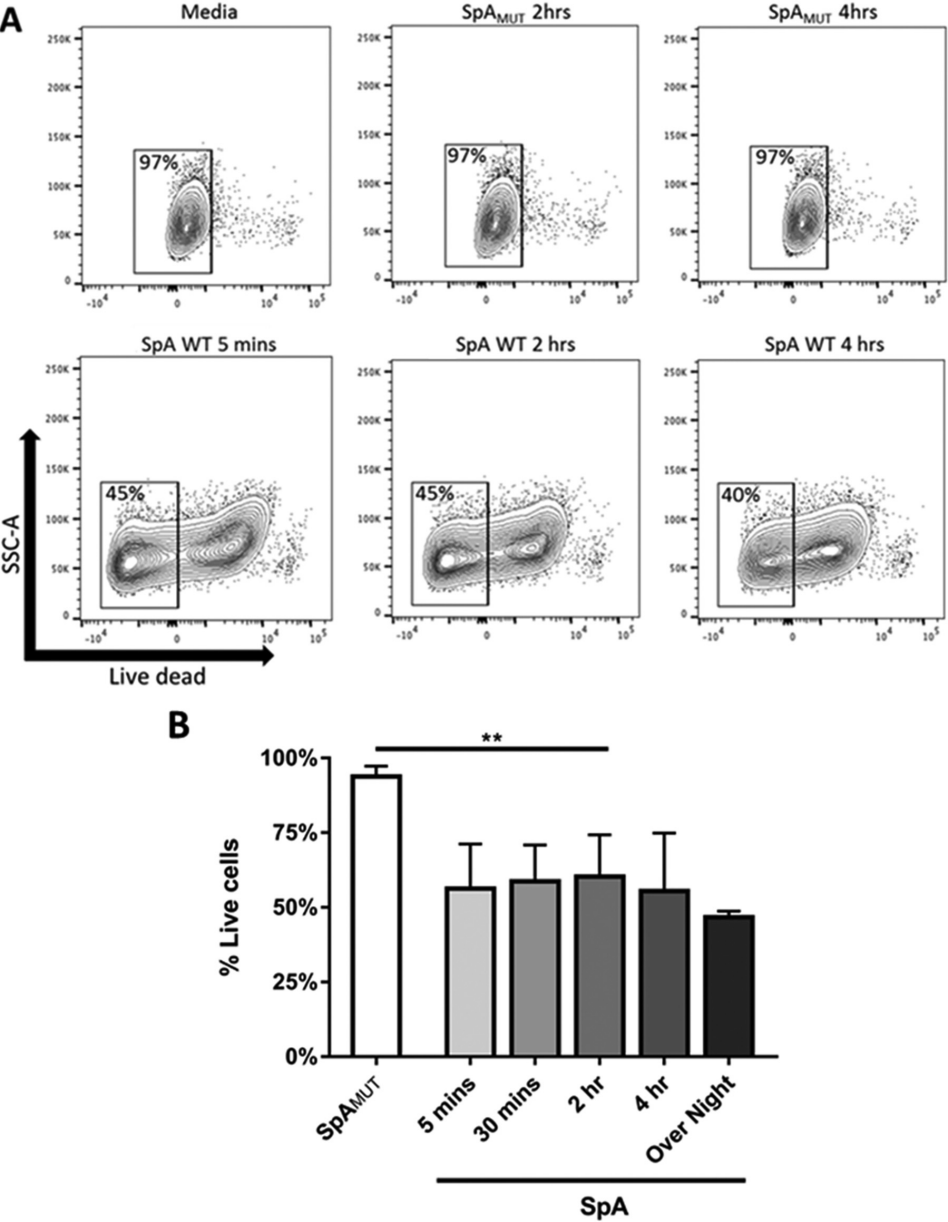
SpA is toxic to purified B cells in the presence of human serum. Purified human B cells (5 × 10^5^) were cultured in media supplemented with 10% human serum in the presence of either 1 μM SpA or SpA_MUT_. At the indicated time points, cells were collected and live cells were identified by flow cytometry as morphologically distinct single cells that were negative for fixable near-IR dead-cell stain. Data shown are representative contour plots (A) and a histogram showing mean and standard deviations (SD) of the percentage of live cells (B). *n* = 2 to 4 independent donors. Data were analyzed using a one-way ANOVA and Dunnett’s multiple-comparison correction. **, *P* < 0.005.

To recapitulate the data obtained from the MAVER-1 cells on primary human cells, SpA’s toxicity was also tested on purified human B cells. B cells were purified via EasySep purification to >90% purity, cultured for up to 16 h (overnight) in 10% (vol/vol) human serum with or without SpA or SpA_MUT_, and analyzed by flow cytometry for changes in viability. SpA_MUT_ in the presence of human serum had no effect on purified B cell viability at any time point tested. Similarly to MAVER-1 cells, however, purified B cells demonstrated significant toxicity within 5 min when cultured in the presence of SpA and human serum (Fig. 3). Interestingly, the scale of this SpA-mediated toxicity was not as acute as that seen with the MAVER-1 cells (mean, 38% cellular loss across all time points); this may be related to the expression of the V_H_3 receptor, which is present on all MAVER-1 cells but whose presence can vary on human B cells.

### The role of complement, antibody isotype, and structure in mediating SpA toxicity.

**FIG 4:**
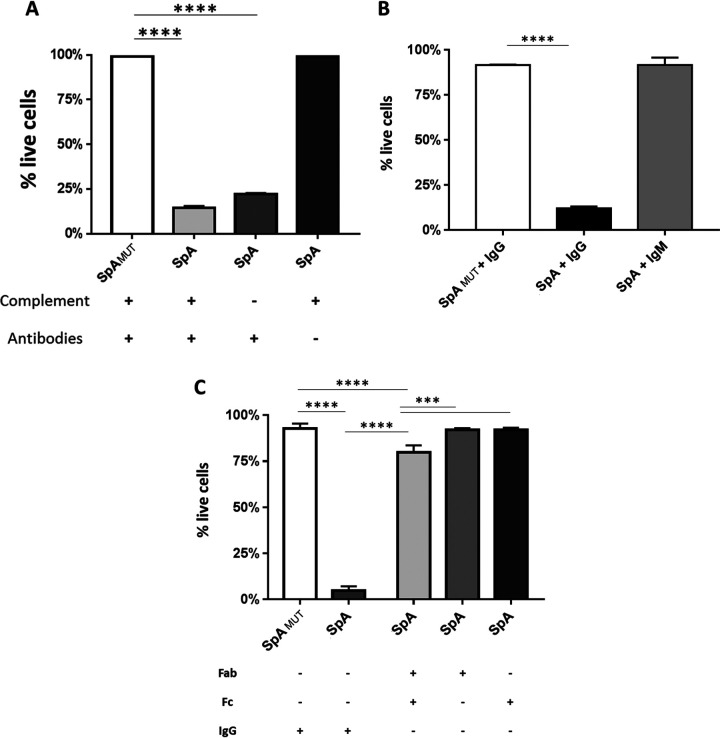
SpA is toxic in the presence of intact IgG only. (A) MAVER-1 cells (5 × 10^5^) were cultured for 2 h in media containing 1 μM SpA or SpA_MUT_ and supplemented with either 10% (vol/vol) standard human serum, heat-inactivated human serum (Complement –), or human serum stripped of antibodies using a protein G column. (B and C) Cells were also cultured for 2 h in PBS containing either 1 μM SpA or 1 μM SpA_MUT_ with and without human IgG or IgM (B) or human IgG Fab, IgG Fc, or an equal mix of both Fab and Fc (C). Live cells were identified by flow cytometry as morphologically distinct single cells that were negative for fixable near-IR dead-cell stain. *n* = 3 to 6 independent experiments. Data were analyzed by one-way ANOVA using the Bonferroni multiple-comparison correction. ***, *P* < 0.0005; ****, *P* < 0.0001.

It has previously been shown that SpA can form immunomodulatory immune complexes with human Ig. As immune complexes have the potential to activate complement via the classical pathway, the importance of complement to the previously described toxicity was investigated. To differentiate between antibody- and complement-mediated effects, human serum was either heat inactivated to deactivate complement or stripped of its antibody fraction using a protein G column. As had been seen previously, SpA_MUT_ demonstrated no toxicity under any conditions. In the presence of untreated human serum, SpA was again toxic, while heat inactivation of the serum had no effect on toxicity. In contrast, depletion of the serum’s immunoglobulin component using a protein G column completely diminished the observed toxicity (Fig. 4A), suggesting that it was the interplay between SpA and human antibodies that caused the cellular toxicity.

The naive B cell receptor is normally comprised of a membrane-bound IgM molecule and associated signaling apparatus, while the most highly expressed isotype of antibodies is the IgG fraction. Thus, the relative influence of these two key subclasses in conferring SpA-mediated toxicity was investigated. MAVER-1 cells were stimulated in serum-free conditions with either SpA or SpA_MUT_ with and without purified human IgM or IgG. SpA demonstrated no significant toxic effects in the presence of IgM, whereas in the presence of IgG, SpA was profoundly toxic (mean, 80% cell viability loss) (Fig. 4B). Cells treated with the SpA_MUT_ in the presence of IgG demonstrated no significant toxicity.

As SpA can bind to both the Fc and the Fab domain of human IgG, it was important to establish whether one interaction is more responsible for the observed toxicity. To this end, MAVER-1 cells were stimulated with SpA in the presence of intact human IgG, purified human IgG Fab, purified human Fc, and an equimolar solution of both Fab and Fc mixed. In the presence of intact human IgG, SpA displayed >90% toxicity, and in the presence of the mixed human Fab and Fc regions, toxicity was also significant (median 10% toxicity); in the presence of the individual components, there were no toxic effects (Fig. 4C). These data indicate that SpA’s toxigenic effects require the availability of full-length IgG, as it is a necessary component for the formation of immune complexes.

### SpA in the presence of human IgG induces rapid necrosis.

**FIG 5:**
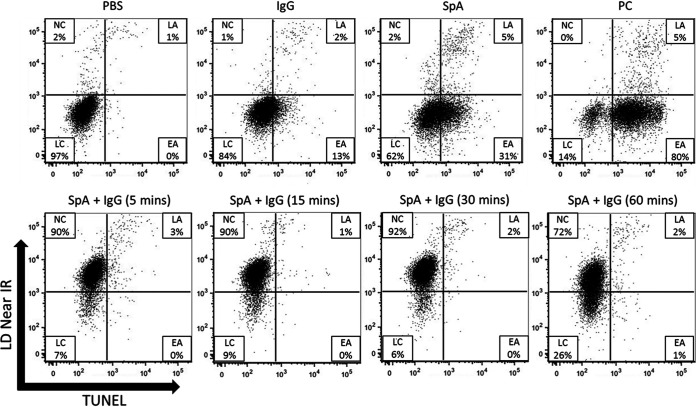
SpA stimulation in the presence of human IgG leads to necrosis. MAVER-1 cells (5 × 10^5^) were cultured for up to 1 h in PBS and treated with 1 μM SpA alone, intact human IgG, and SpA plus human IgG or DNase (positive control [PC]). The samples were then analyzed using a Click-iT TUNEL flow cytometry assay system for assessing the number of live cells (LC), early-apoptosis cells (EA), late-apoptosis cells (LA), and necrotic cells (NC). Cells were tracked from 5 to 60 min. Cells were permeabilized and incubated with DNase for the purpose of generating the positive control. Representative results are from 2 individual experiments.

To establish the mechanism of this IgG-dependent cell toxicity, MAVER-1 cells were stimulated with SpA in the presence of purified IgG in serum-free conditions from 5 min to 60 min, and the cells were then analyzed for apoptotic cell death using a terminal deoxynucleotidyltransferase-mediated dUTP-biotin nick end labeling (TUNEL) Click-iT apoptosis staining assay. Given that cross-linking of the mouse BCR by SpA has previously been shown to deplete B cell populations *in vivo* (26), it was expected that this depletion was the result of SpA-induced cellular activation, followed by cellular anergy and eventual clonal collapse (8, 41). It was, therefore, anticipated that a significant proportion of cells would become apoptotic prior to necrosis. However, within just 5 min of stimulation with SpA and human IgG, 90% of cells stained positive for LIVE/DEAD fixable dead-cell stain and negative for TUNEL staining, indicating that they were necrotic as opposed to apoptotic (Fig. 5). SpA treatment in the absence of IgG led to an increase in apoptotic signals, with about 31% of cells staining TUNEL positive after 60 min, indicating that in the absence of human IgG, SpA may act to induce apoptosis, as had been reported previously for mice.

### Immune sera from mice vaccinated with SpA_MUT_ protects MAVER-1 cells from SpA-mediated toxicity.

Having established SpA’s toxigenic effects on human cells, we wanted to investigate if antibodies raised through vaccination could inhibit these effects. To this end, the *in vitro* protective capacity of mouse sera raised through vaccination with an adjuvanted solution of SpA_MUT_ was tested on MAVER-1 cells. BALB/c mice were vaccinated twice, 30 days apart, with either an adjuvanted solution containing SpA_MUT_ or the adjuvant alone. Serum samples were collected from these mice 21 days after the second immunization and pooled. These sera were then incubated for 30 min in the presence of both SpA and purified human IgG, before addition to MAVER-1 B cells and incubation for 15 min. To maximize the chance that an effect might be observed and to allow for the greatest relative concentration of mouse sera, reduced concentrations of both SpA and human IgG were used in comparison to the concentrations used in earlier experiments.

**FIG 6:**
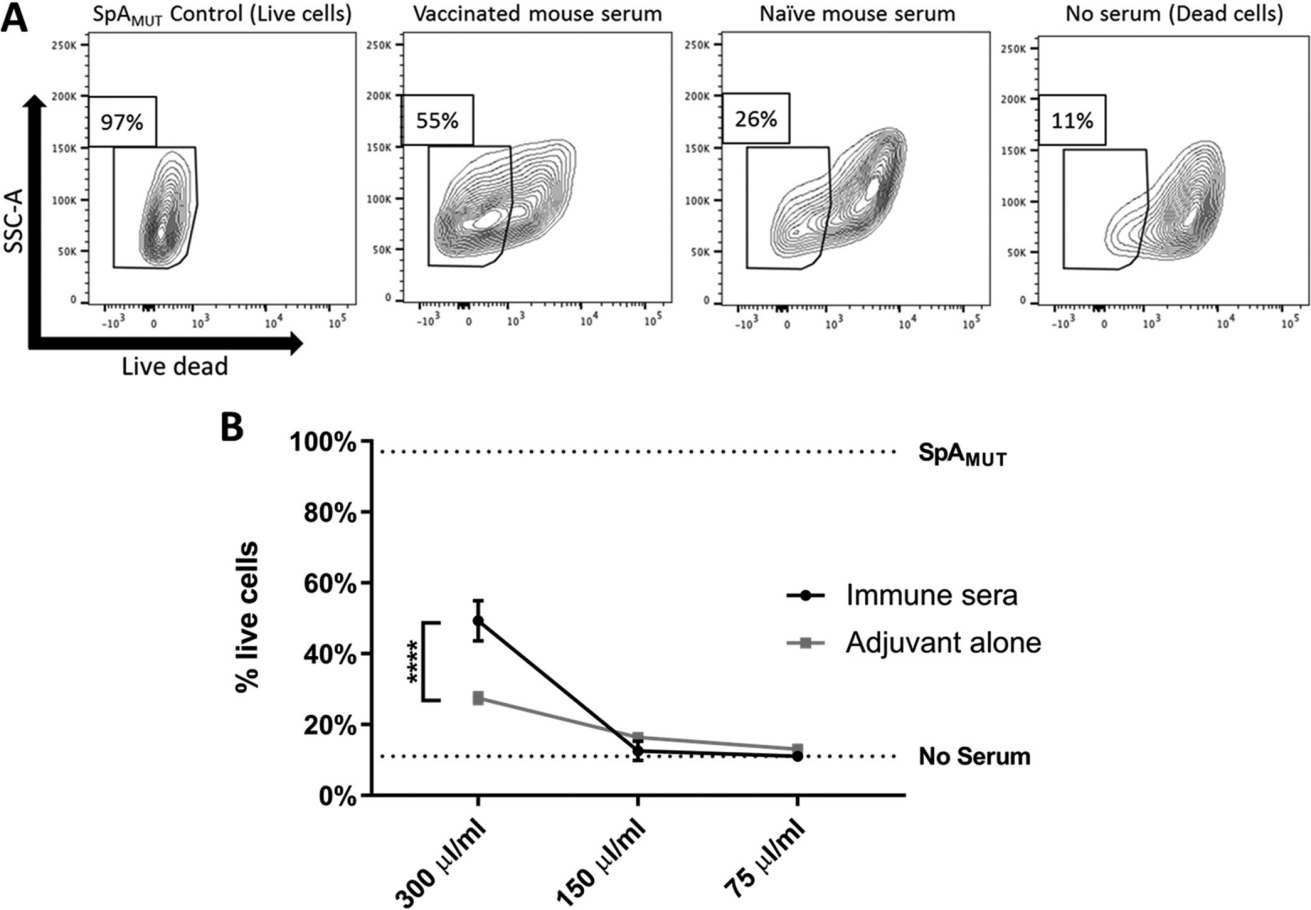
Serum derived from SpA_MUT_-vaccinated mice protects MAVER-1 cells against SpA-mediated toxicity. Sera were isolated from BALB/c mice that had received two doses of either a vaccine formulation containing SpA_MUT_ or the adjuvant alone 30 days apart. Various concentrations of sera from these mice were incubated for 1 h in PBS containing 100 μM SpA and 75 μg/ml human IgG. To this solution, 5 × 10^5^ MAVER-1 cells were added for 15 min. Live cells were then identified by flow cytometry as morphologically distinct single cells that were negative for fixable near-IR dead-cell stain; data shown are representative contour plots (A) and percentages of live cells (B). *n* = 3. Data were analyzed by a two-way ANOVA using Sidak’s multiple-comparison correction. ****, *P* < 0.0001.

The sera raised through vaccination with the adjuvanted SpA_MUT_ provided significant protection to MAVER-B cells (mean, 49% survival) compared to that of cells treated with SpA plus purified IgG in the absence of immune sera (mean, 10% survival) (Fig. 6). The sera generated from the control group vaccinated with adjuvant alone provided some protection, but this was not significantly increased above that of the control group. As expected, MAVER-1 cells cultured with SpA_MUT_ instead of SpA showed no loss in viability (97% survival).

### SpA toxicity is not limited to B cells.

**FIG 7:**
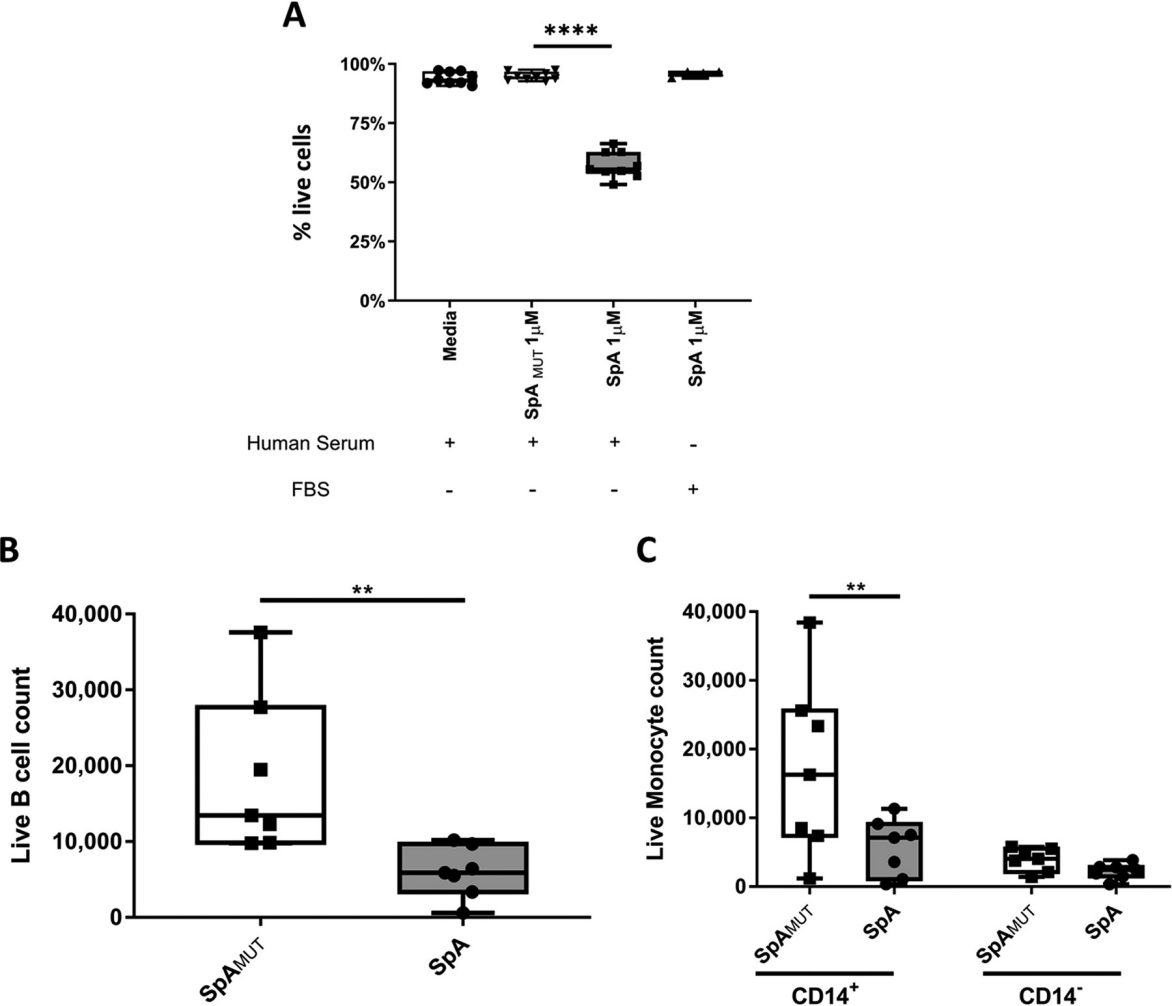
SpA is toxic to human primary leukocytes in the presence of human serum. Human PBMCs (1.5 × 10^6^) were cultured overnight in media supplemented with 10% FBS or human serum in the presence of either SpA or SpA_MUT_. Live cells were identified by flow cytometry as morphologically distinct single cells that were negative for fixable near-IR dead-cell stain. (A) Results were obtained from 3 to 4 independent donors. Cells were then analyzed for their expression of phenotypic markers by flow cytometry. (B and C) B cells were identified as CD3^−^ CD19^+^ (B), monocytes were identified as CD3^−^ CD19^–^ CD56^−^ HLA-DR^+^ CD14^+^, and dendritic cells were identified as CD3^−^ CD19^–^ CD56^−^ HLA-DR^+^ CD14^−^ (C). Data were analyzed by one-way ANOVA using Dunnett’s multiple-comparison correction (A), by the Mann-Whitney test (B), or by two-way ANOVA using Sidak’s multiple-comparison correction (C). **, *P* < 0.005; ****, *P* < 0.0001.

To establish if the cytotoxic effects induced by SpA in the presence of IgG on B cells would also occur on other primary leukocytes, PBMCs were isolated from healthy donors and stimulated with either SpA or SpA_MUT_ in medium supplemented with either 10% human serum or 10% FBS for 16 h. In line with our previous findings, no toxic effects were observed in the total leukocytes stimulated with SpA_MUT_ or those stimulated with SpA in FBS, while leukocytes stimulated with SpA in the presence of human serum displayed an approximately 40% loss of cellular viability (Fig. 7A). As monocytes are involved in the sensing and clearance of immune complexes, we sought to establish if they too were affected by SpA’s toxigenic effects. To that end, the stimulated PBMCs were phenotyped for both B cell and monocyte populations by flow cytometry, and viability was again analyzed via LIVE/DEAD fixable dead-cell stain. Both CD19^+^ B cells and HLA-DR^+^ CD14^+^ monocytes showed significant losses in cellular viability when treated with SpA in the presence of human serum, while HLA-DR^+^ CD14^−^ dendritic cells showed no significant change in viability (Fig. 7B and C). These data indicate that SpA-mediated toxicity is not limited to BCR-expressing cells, as it has been previously theorized. Monocyte susceptibility may be due to their greater expression of certain Fc receptors, as these receptors can be involved in sensing and responding to immune complex formation.

## DISCUSSION

One of the greatest impediments to the generation of an efficacious anti-S. aureus vaccine has been the intrinsic ability of the bacterium to evade the adaptive immune response and the subsequent lack of a definitive clinical natural correlate of protection (5, 6, 42). SpA has long been established as a key S. aureus virulence factor involved in overcoming the adaptive humoral immune response (12, 29, 38). The question of how SpA influences the humoral response has raised numerous answers stemming from its plethora of interactions and effects within the B cell compartment. Herein, we have confirmed that these interactions and resulting effects are dependent on the human-specific environment. Furthermore, our work demonstrates that SpA’s role as a human B cell toxin is dependent upon the presence of full-length human IgG and that this toxicity can be inhibited by anti-SpA serum raised in mice through vaccination with a nonbinding SpA mutant. Additionally, we have shown that this toxicity affects cells beyond the B cell compartment and is thus not entirely reliant on the SpA-BCR interaction, as has been previously hypothesized (Protalex). These findings may help further elucidate SpA’s effect on the human humoral immune response and thus in the rational design of an efficacious anti-S. aureus vaccine.

Our results have demonstrated that the human B cell line MAVER-1 is activated in response to direct stimulation with SpA. Importantly, it was noted that FBS acts to inhibit this activating interaction. As FBS is overwhelmingly the most common serum used during human *in vitro* cell culture experiments, its inclusion may act to obfuscate the true extent of SpA’s role as a virulence factor. Indeed, we observed that the use of human serum during cell culture showed a heretofore-unseen degree of toxicity on both MAVER-1 cells and, importantly, on primary human B cells at a concentration of SpA that has been shown to be biologically relevant in mouse abscess models (32), indicating that the underlying mechanism of action is an intrinsic feature of SpA’s interaction with the human immune system in general and human serum specifically. To better explore this finding, we chose to focus on SpA’s effects on human cells in the presence of human serum and human serum components.

It has been hypothesized that one of SpA’s major roles as a virulence factor is the dysregulation of the humoral immune response through inappropriate activation of naive V_H_3^+^ B cells, resulting in their clonal proliferation, anergy, and apoptotic collapse. To test this superantigen-induced apoptosis model, we used a novel Click-iT-based TUNEL assay to track DNA fragmentation in our culture system across several time points. It was found that the MAVER-1 B cells showed membrane disruption within the first 5 min of coculture but no DNA fragmentation. Thus, it can be inferred that SpA’s toxicity in this case was not dependent upon the activation of the MAVER-1 B cells, as would be expected in a superantigen-induced model. This rapid induction of membrane disruption by SpA in the presence of human IgG indicates that the observed toxic effects are immune complex mediated, a finding that we furthered by demonstrating that complement, the other major immune component of human serum, seemingly played no role in this process. As such, any attempt to neutralize SpA as a toxin may not be dependent upon our ability to block its interaction with the BCR but rather to prevent complex formation with IgG.

**FIG 8:**
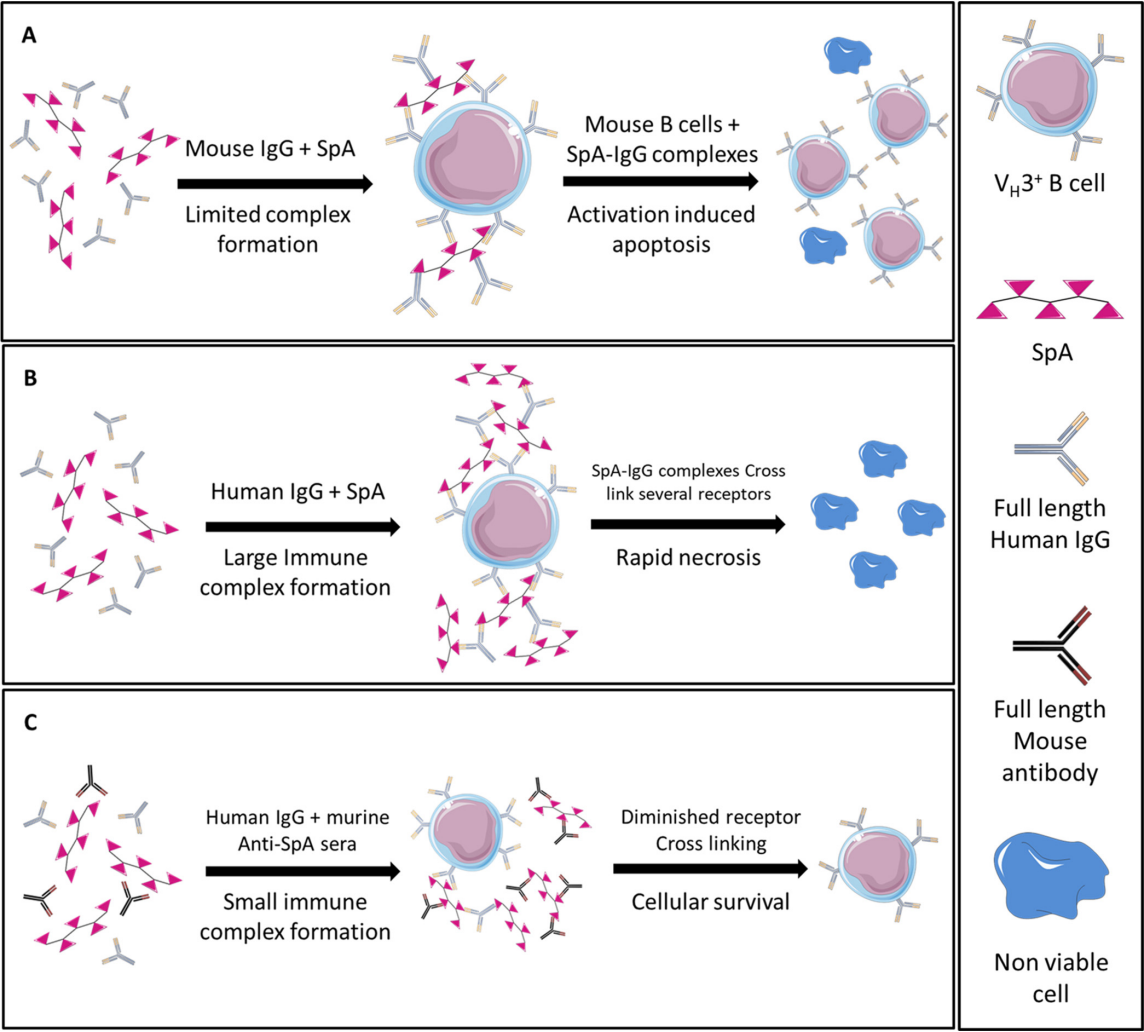
Proposed mechanism of action for SpA-induced toxicity. (A) In the presence of murine IgG, SpA forms limited immune complexes; these smaller complexes cross-link the BCR on murine V_H_3^+^ B cells, leading to clonal expansion and eventual collapse via apoptosis of these cells. (B) In contrast, in the presence of full-length human IgG, SpA forms large immune complexes; these complexes then interact with V_H_3^+^ B cells, leading to rapid necrosis of these cells. (C) The presence of murine anti-SpA sera prevents the formation of large immune complexes and thus acts to limit SpA-mediated toxicity.

Treatment of MAVER-1 B cells with SpA alone (without human IgG) led to an increase in apoptotic signals; this may indicate that in the absence of human IgG, SpA may act to induce apoptosis, as has been reported previously in mice (5). Importantly, mouse immunoglobulin has a low affinity for SpA and may not fully recapitulate the pathogenic mechanisms of SpA in humans. Therefore, we propose a mechanistic model in which, in humans, SpA is expected to induce cell death via necrosis but in which, in mice, supraclonal collapse is caused by activation-induced apoptosis (Fig. 8). SpA is a remarkably flexible molecule, which allows it to bind multiple antibodies via either the Fc or the Fab domain without becoming sterically hindered (25, 43). This promiscuity in binding partners raises an issue in identifying the relative importance of each individual interaction. To address this, SpA was cultured with either intact human IgG or purified human IgG Fab and Fc individually or in an equimolar mixture. Maximal toxicity required intact IgG; the equimolar mixture of Fab and Fc fragments did demonstrate some limited toxicity, but the individual components displayed no toxicity. This reliance on intact IgG further indicates that SpA’s toxicity is reliant upon the formation of immune complexes through the formation of SpA-IgG lattices. The immunogenicity of such SpA immune complexes has previously been observed in both mice and rabbits (31, 32, 44). Thus, we hypothesize that SpA in the presence of full-length human IgG forms macro-lattices through its interactions with both the Fc and Fab regions of immunoglobulins. These lattices then interact with any available cognate receptors, such as the BCR, and act to cross-link them, leading to membrane disruption during internalization of the affected receptors. Variation in SpA expression across S. aureus strains has been observed (13); thus, we might likewise expect to find variation in the rates of immune complex formation.

Immune complex interactions are, however, not solely limited to B cells, and thus we hypothesized that SpA-mediated immune complexes may be capable of broader interactions than had previously been observed. Indeed, our work shows that SpA’s toxicity extends beyond B cells specifically, as it also shows considerable toxicity toward monocytes. This provides additional support to the theory that SpA’s observed toxicity is not solely reliant upon the interaction with the V_H_3^+^ B cell receptor. Interestingly though, when purified primary human B cells were treated with SpA in the presence of human serum, we observed only an ∼50% loss in viability, which is in keeping with the expected level of V_H_3 positivity. This may indicate that while the V_H_3 B cell receptor is not solely required, it is still the primary target for SpA on B cells.

As discussed earlier, the development of an anti-S. aureus vaccine has been inhibited by the inability to generate an efficacious humoral immune response, due in part to SpA. Thus, the disruption of SpA’s role as a virulence factor may be a key step in the generation of an effective S. aureus vaccine, but given SpA’s ability to indiscriminately bind broad families of antibodies, it is unknown whether anti-SpA antibodies can inhibit its biological functions. To address this question, we generated anti-SpA sera in mice through vaccination with a SpA mutant; using these sera, we provided significant protection to MAVER-1 cells from SpA-mediated toxicity. These data indicate that murine anti-SpA antibodies can overcome SpA’s intrinsic ability to sequester them. The neutralization of SpA in this case is most likely the consequence of high-avidity anti-SpA murine antibodies preferentially binding to SpA and thus displacing the nonspecific human immunoglobulins; further research is required to fully identify the nature of this interaction.

In conclusion, our study has outlined a novel interaction between SpA and human immune components which may help guide rational vaccine creation. We have further demonstrated the need to study SpA and S. aureus using humanized systems in order to identify protective effects. Indeed, overcoming SpA’s disruptive effects on the humoral immune response may require either (i) antibodies against staphylococcal antigens in titers that are high enough to overwhelm available SpA binding sites and complexes (in an excess sufficient to opsonize and neutralize the underlying S. aureus infection) or (ii) anti-SpA antibodies in quantities that are sufficient to prevent complex formation and to opsonize the cell wall-bound SpA present on the bacterium. Ultimately, an efficacious S. aureus vaccine may need to use both stratagems.

## MATERIALS AND METHODS

### SpA and SpA_MUT_ expression.

Escherichia coli cells expressing histidine-tagged nonmutated SpA (SpA) or SpA_MUT_ (a mutated variant of SpA which contains single amino acid substitutions [10] within the five immunoglobulin binding sites that abolish SpA’s interactions with both the Fc and Fab antibody domains and the V_H_3^+^ BCR) were collected by centrifugation and disrupted by ultrasonic oscillation. The proteins were then isolated and purified using immobilized metal affinity chromatography (IMAC), ceramic hydroxyapatite (CHT), and size exclusion chromatography, respectively. Protein purity and molecular weight were analyzed by size exclusion ultrahigh-performance liquid chromatography (SE-UPLC), and proteins were found to have >85% purity. Samples were further analyzed for endotoxin content and found to have <0.1 endotoxin unit (EU)/μl.

### MAVER-1 cell line.

The MAVER-1 human mantle cell lymphoma B cell line was purchased from the American Type Culture Collection (ATCC CRL-3008). Cells were cultured in RPMI 1640 medium supplemented with 10% fetal bovine serum (FBS) in a range of ≥3 × 10^5^ to ≤2 × 10^6^ cells/ml. Cells were split into fresh medium every 2 to 3 days. The cells were disposed after a maximum of 25 passages. Before use, the cells were washed twice in warm Dulbecco’s phosphate-buffered saline (DPBS), resuspended at the required concentration, and placed into a 96-well U-bottom plate (Corning).

### PBMC isolation.

Blood donor buffy coats were obtained from the Hospital of San Giuseppe di Empoli, Tuscany, Italy. PBMCs were isolated by diluting the buffy coats 1:1 in Hanks’ balanced salt solution (HBSS). Ficoll density gradient separation was used to separate the cells by spinning the cells at 800 × *g* for 30 min at room temperature in a 50-ml Falcon centrifuge tube containing up to 30 ml of buffy coat loaded over 15 ml of Ficoll. The stratified leukocytes were transferred to fresh Falcon tubes and washed with HBSS. The viable cells were then enumerated using trypan blue, and the PBMCs were frozen in complete RPMI (cRPMI) medium containing 10% dimethyl sulfoxide (DMSO). cRPMI medium is comprised of RPMI 1640 (Gibco), 10% (vol/vol) fetal calf serum (HyClone), 1% (vol/vol) 100× penicillin-streptomycin-glutamine (EuroClone), 1% (vol/vol) nonessential amino acids (Gibco), and 1% (vol/vol) Na pyruvate (Gibco).

### B cell isolation.

Frozen PBMCs were thawed at 37°C in a thawing solution which consisted of PBS, 2.5 mM EDTA, and 20 μg/ml DNase and resuspended in cRPMI medium. Viable cells were enumerated using a Beckman Coulter Vi-cell XR cell counter. The cells were then resuspended at 5 × 10^7^ cells/ml and then separated using the EasySep human B cell isolation kit per the manufacturer’s protocol. Isolated B cells were resuspended in RPMI 1640 medium containing 10% (vol/vol) human serum and placed at 5 × 10^5^ cells/well in a 96-well plate.

### MAVER-1 activation and toxicity assays.

MAVER-1 cells were resuspended at 5 × 10^5^ cells/well in 100 μl RPMI 1640 medium with and without 1% or 10% (vol/vol) FBS, 10% (vol/vol) human serum (Euro Clone), or PBS and with and without human IgG (500 nM), human IgG Fab (2 μM), human IgG Fc (4 μM), and IgM (50 nM); they were then stimulated with 1 μM SpA or SpA_MUT_ for 2 h at 37°C (all purified human antibodies were purchased from Sigma-Aldrich). The complement in human serum was inactivated by heating the sample in a water bath to 56°C and holding it there for 30 min. HiTrap (1-ml) protein G columns (GE Healthcare) were used as per the manufacturer’s protocol to remove the immunoglobulin component of human serum.

### SpA neutralization assay.

The inhibition of SpA-induced toxicity by functional anti-SpA antibodies was evaluated in an *in vitro* assay. Serial dilutions of sera from immunized mice were incubated with 1 μM SpA and 1 μM IgG for 30 min at 37°C. MAVER-1 cells (5 × 10^5^) were then added, and the incubation was prolonged for an additional 15 min at 37°C.

### Apoptosis assay.

MAVER-1 cells were resuspended at 5 × 10^5^ cells/well in 100 μl PBS containing human IgG (500 nM) with and without 1 μM SpA or SpA_MUT_ and incubated for various time points at 37°C in 5% CO_2_. Following stimulation, the cells were washed twice in DPBS, stained for 20 min on ice with LIVE/DEAD fixable dead-cell stain (Life Technologies), and fixed using Cytofix (BD), and the cells were then washed and resuspended for 30 min in DPBS containing 1% bovine serum albumin (BSA) and saponin; following this, the cells were treated with the TUNEL (Roche) TdT reaction kit as per the manufacturer’s instructions. The cells were then stained using the Click-iT reaction mix (Alexa-488; Thermo Fischer) as per the manufacturer’s instructions. Finally, the cells were washed in cold PBS and immediately acquired on a special-order system (SOS) flow cytometer (BD Biosciences).

### Mouse immunization protocol.

Female BALB/c mice were purchased from Charles River Laboratories. Six-week-old mice were used for all experiments. Mice were immunized intramuscularly (i.m.) with 10 μg of SpA_MUT_ in PBS, formulated with adjuvant in a total volume of 50 μl (25 μl/quadriceps). Control mice received an equal volume of adjuvant. Mice were given two doses 30 days apart and bled 3 weeks after the second dose.

### PBMCs and purified B cell toxicity assays.

Frozen PBMCs were thawed at 37°C in a thawing solution which consisted of PBS, 2.5 mM EDTA, and 20 μg/ml DNase. The PBMCs were centrifuged at 350 × *g* for 5 min at room temperature and resuspended in cRPMI medium. Viable cells were enumerated using a Beckman Coulter Vi-cell XR cell counter. The cells were then resuspended at 1.5 × 10^6^ cells per 100 μl. One hundred microliters of the cell solution was then added to a pre-prepared 96-well plate that contained the relevant stimuli at 2× their final concentration in cRPMI medium. The samples and stimuli were incubated overnight at 37°C in 5% CO_2_.

The prepared isolated human B cells were resuspended in RPMI 1640 medium containing 10% (vol/vol) human serum and placed at 5 × 10^5^ cells/well in a 96-well plate. The cells were then stimulated with 1 μM SpA or SpA_MUT_ and incubated for various time points at 37°C in 5% CO_2_.

### Flow cytometry.

PBMCs, purified B cells, and MAVER-1 B cells were prepared as described above. Cells were centrifuged at 350 × *g* for 5 min and washed with warm PBS twice. The cells were then stained for 20 min on ice with LIVE/DEAD fixable viability stain (diluted 1:1,000 in PBS) (Life Technologies), washed in cold DPBS, incubated at room temperature (RT) for 20 min in 2% naive rabbit serum, and then stained 1:1 with appropriate concentrations of previously titrated extracellular antibodies for 20 min. The samples were then washed twice with cold PBS and acquired on an LSRFortessa flow cytometer (BD Biosciences), except in the apoptosis assay, whose samples were analyzed on an SOS flow cytometer (BD Biosciences). Samples were analyzed with FlowJo software, version 9.5 (TreeStar Inc.). Gating for analysis was set on relevant isotype controls for each antibody or on fluorescence minus one (FMO) controls. Antibodies used were CD19 phycoerythrin (PE)-Cy5 (clone HIB19; BD Biosciences), CD69 BUV 737 (clone FN50; BD Biosciences), CD16 BV421 (clone 3G8; BD Biosciences), CD64 PE (clone 10.1 BioLegend), and CD14 BV786 (clone M5E2; BD Biosciences).

### Statistical analysis.

Statistical analyses were performed using GraphPad prism version 7. Differences between normally distributed groups were analyzed using an unpaired *t* test, a paired *t* test, and/or a one-way or two-way analysis of variance (ANOVA) with Tukey’s, Dunnett’s, Bonferroni’s, or Sidak’s multiple-comparison test, where appropriate. Differences between nonnormally distributed groups were analyzed using a Mann-Whitney U test. A *P* value of ≤0.05 was considered significant.

### Ethical statement.

Buffy coats from healthy donors were obtained from the Blood Transfusion Section, Hospital of San Giuseppe di Empoli. Informed consent was obtained before all blood donations. The study protocol conforms to the ethical guidelines of the 1975 Declaration of Helsinki (45). Pooled human sera from a healthy subject were purchased from EuroClone (written informed consent was obtained from each subject in compliance with the World Medical Association Declaration of Helsinki). All animal studies were ethically reviewed and carried out in accordance with European Directive 2010/63/EEC and the GSK policy on the care, welfare, and treatment of animals.
