# Supplementary figures and images for: Staphylococcal Protein A Induces Leukocyte Necrosis by Complexing with Human Immunoglobulins
Source: mBio. 2021 Jun 1;12(3):e00899-21. doi: 10.1128/mBio.00899-21 (PMC8262926; doi:10.1128/mBio.00899-21)

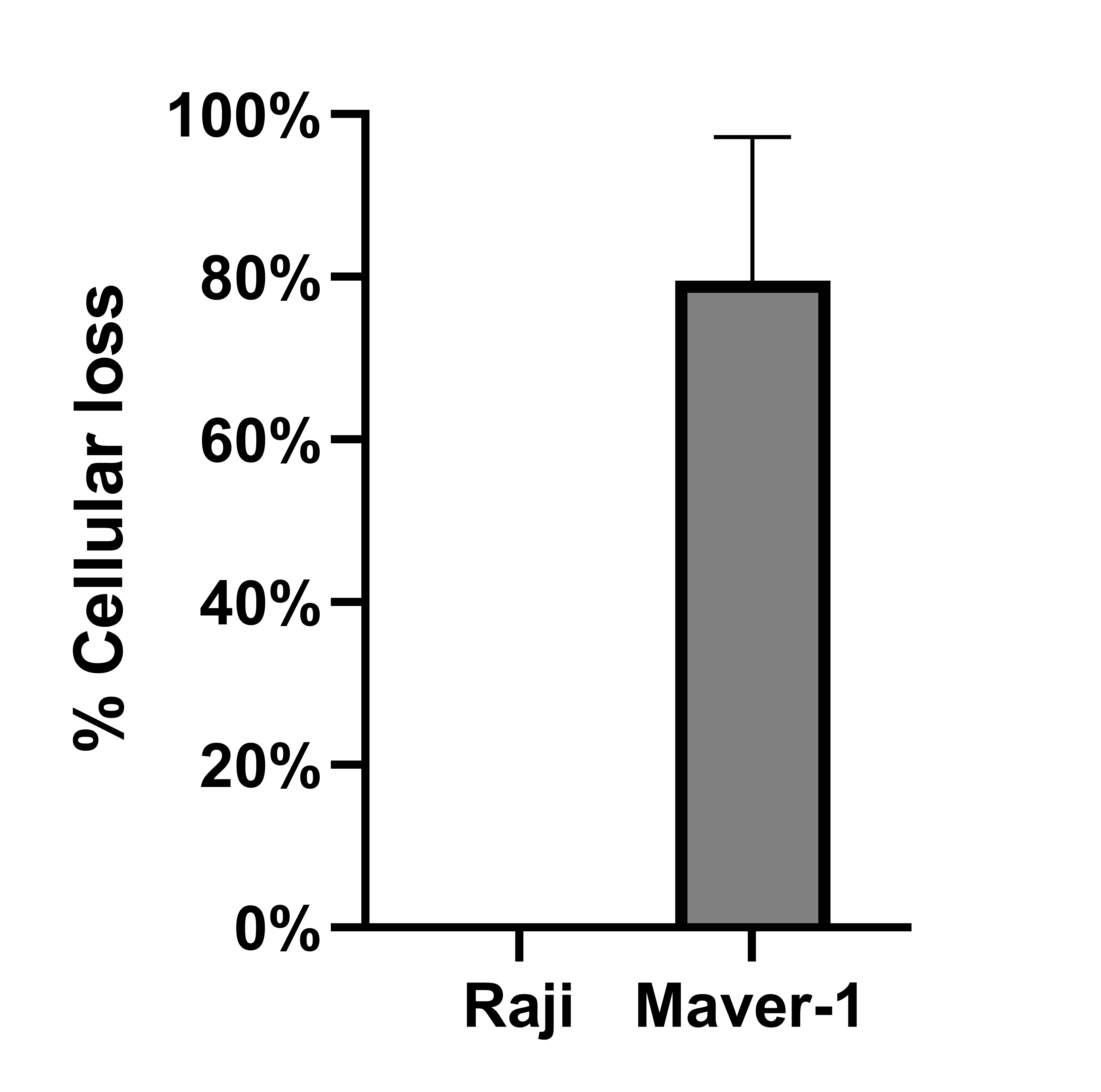

Supplement: FIG S1 [file mbio.00899-21-sf001.tif]
